# EXPRESSION OF CONCERN: A Newly Identified 45‐kDa JAK2 Variant with an Altered Kinase Domain Structure Represents a Novel Mode of JAK2 Kinase Inhibitor Resistance

**DOI:** 10.1002/1878-0261.70294

**Published:** 2026-06-21

**EXPRESSION OF CONCERN**: S. Prasad Gorantla , T. Andreas Mueller , C. Albers‐Leischner , M. Rudelius , N. von Bubnoff , and J. Duyster , “A Newly Identified 45‐kDa JAK2 Variant with an Altered Kinase Domain Structure Represents a Novel Mode of JAK2 Kinase Inhibitor Resistance,” Molecular Oncology 18, no. 2 (2024): 415‐430, 10.1002/1878-0261.13566.38104968 PMC10850816

This Expression of Concern is for the above article, published online on 17 December 2023 in Wiley Online Library (wileyonlinelibrary.com), and has been issued by agreement between the journal Editor‐in‐Chief, Kevin Ryan; the Federation of European Biochemical Societies; and John Wiley and Sons Ltd. Concerns were raised by a third party on PubPeer [1] that the STAT5 band in Fig. 4D had been duplicated in another article by some of the same authors [Gorantla et al. 2025 (https://doi.org/10.1038/s41375-025-02594-7)]. These concerns were confirmed by the editors.

The authors responded to an inquiry by the publisher and presented data to verify the results presented in the article. The authors further stated that research was conducted between 2010 and 2012. The publisher and editors reviewed these data and found much of the data are no longer available or not available in their original form or format. As such, the editors determined that a correction would not be acceptable.

The Expression of Concern has been agreed to inform and alert readers, because the presence of a band that has been duplicated in a later article and presented as different experimental results casts doubt on the accuracy of the reporting of these data in this article.
